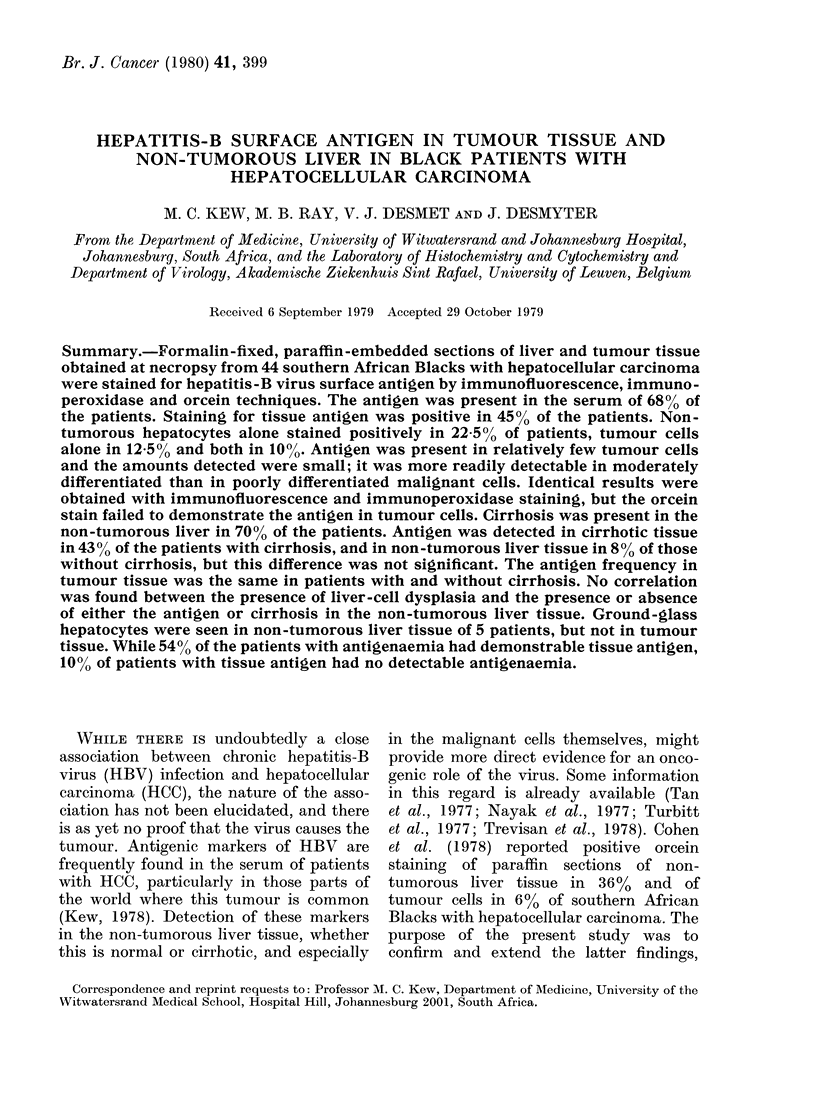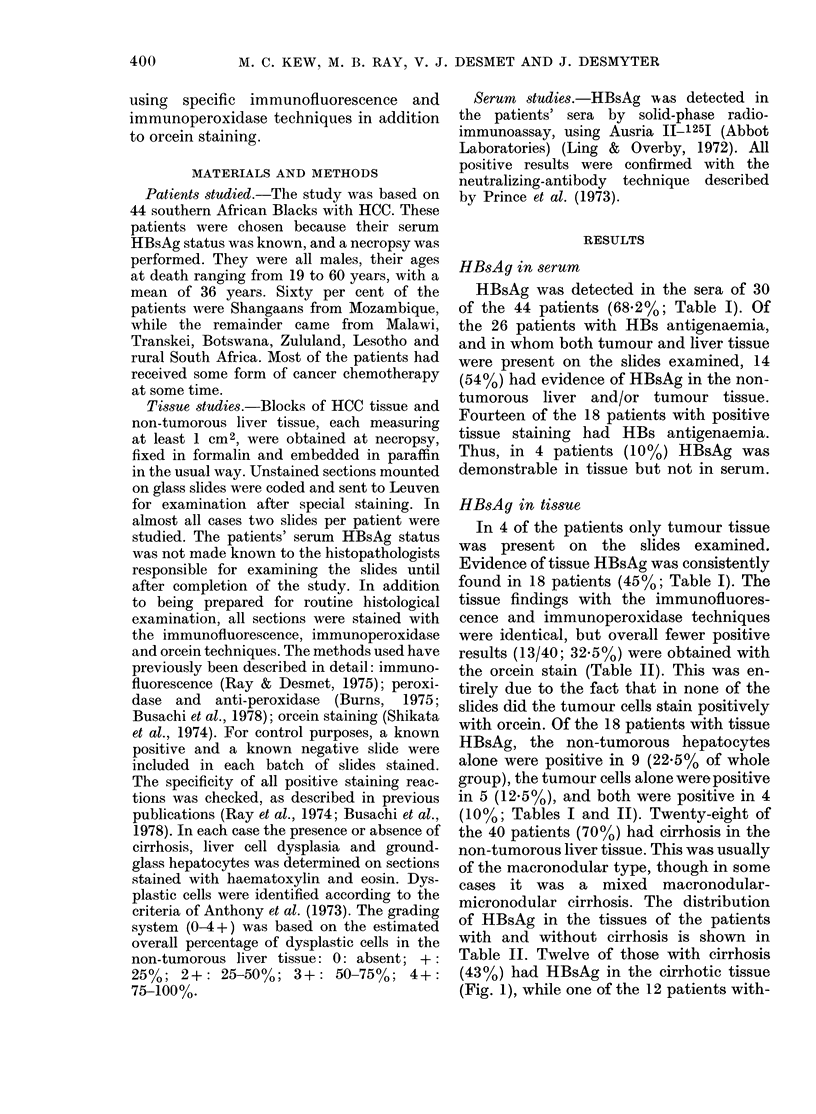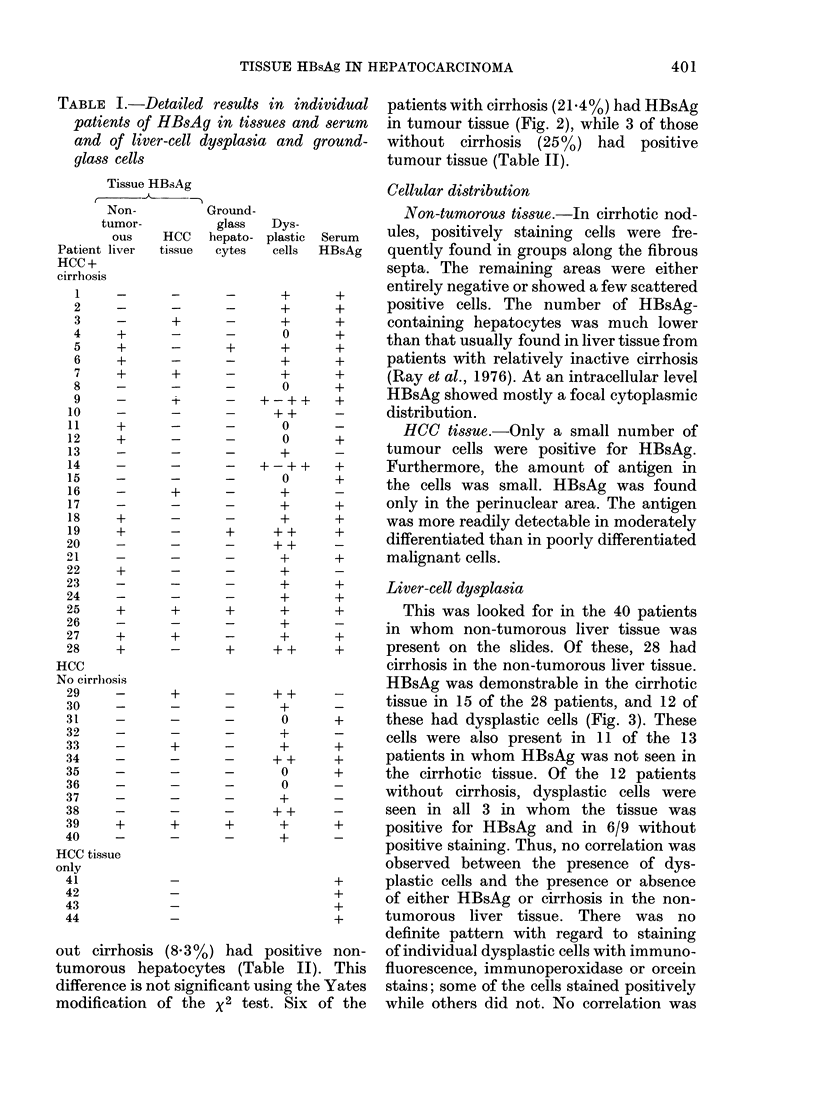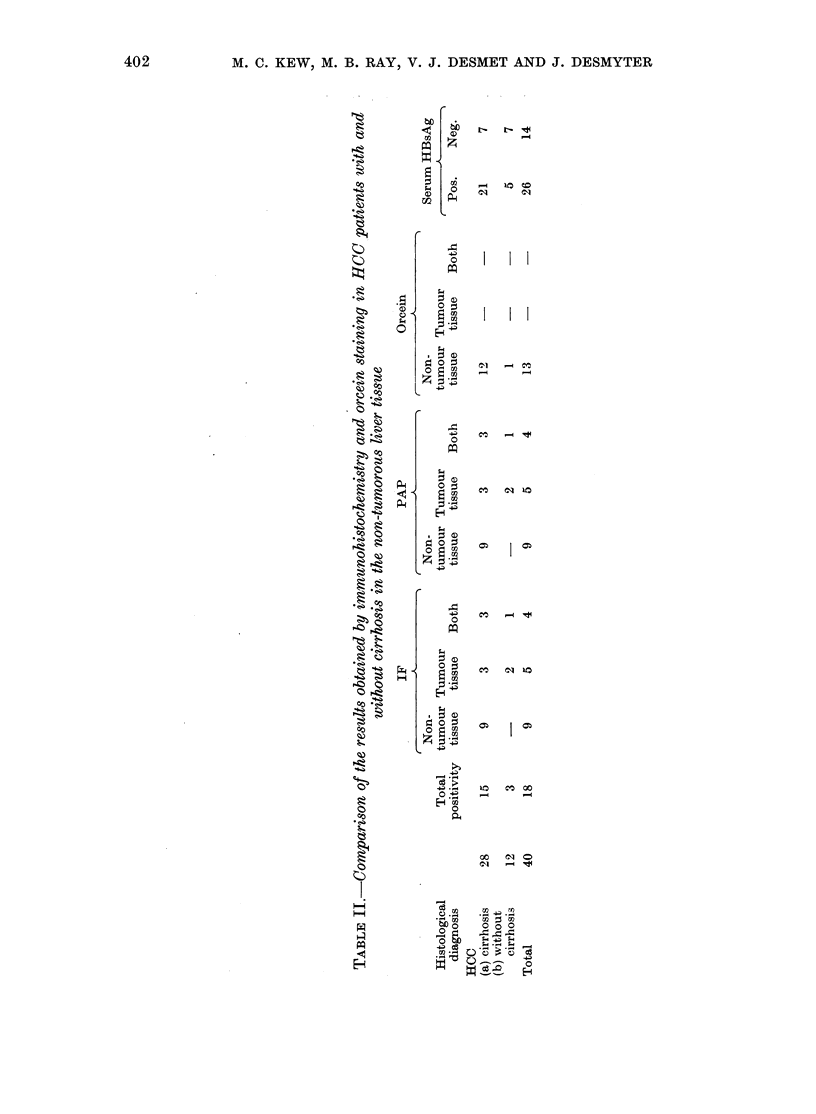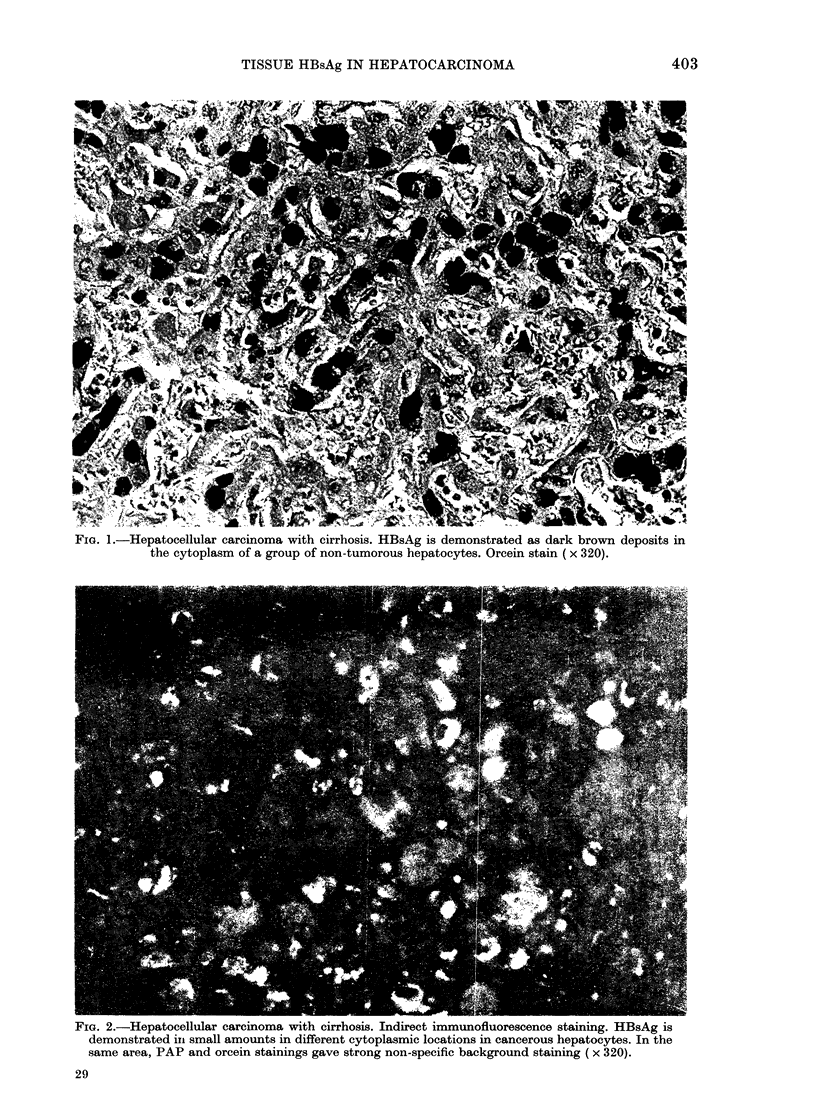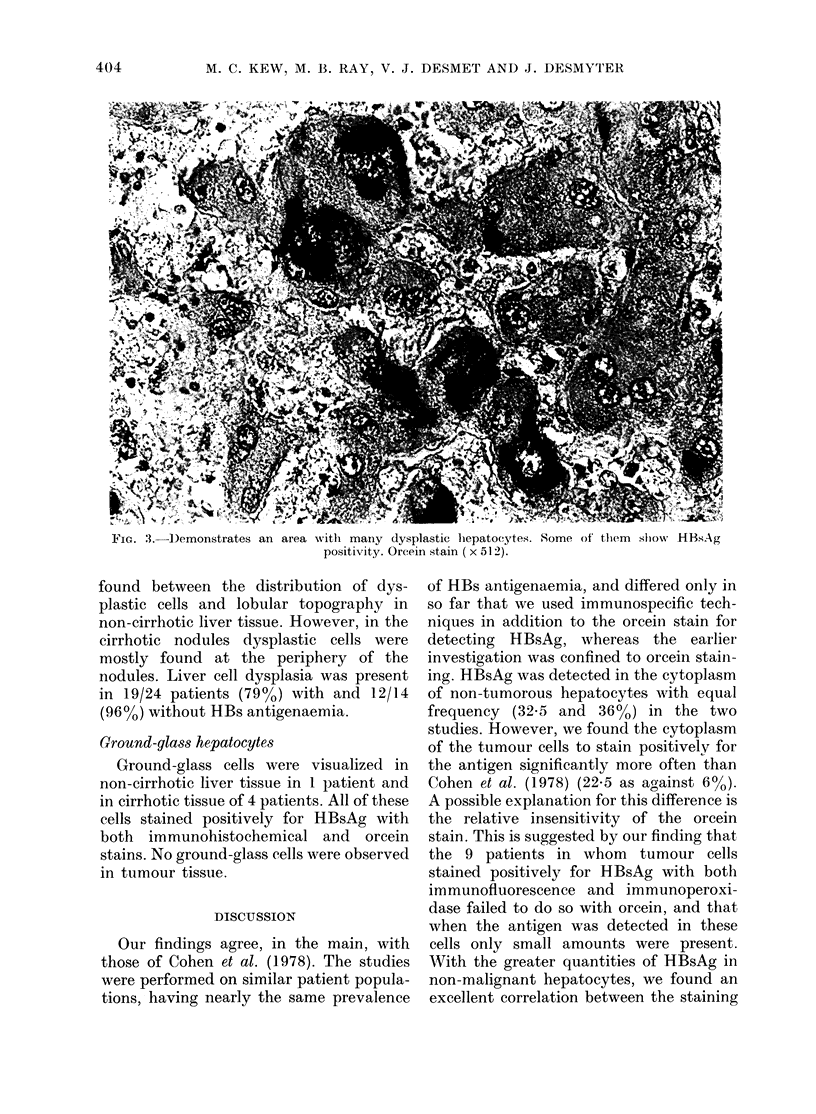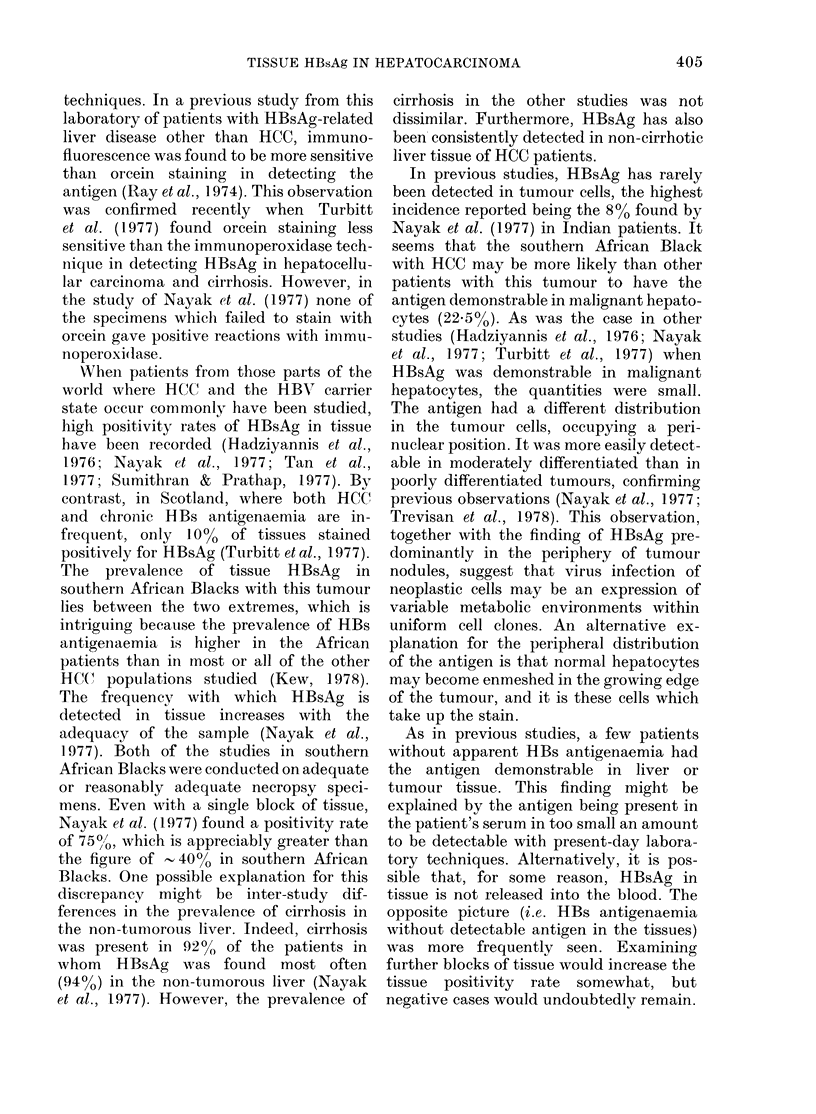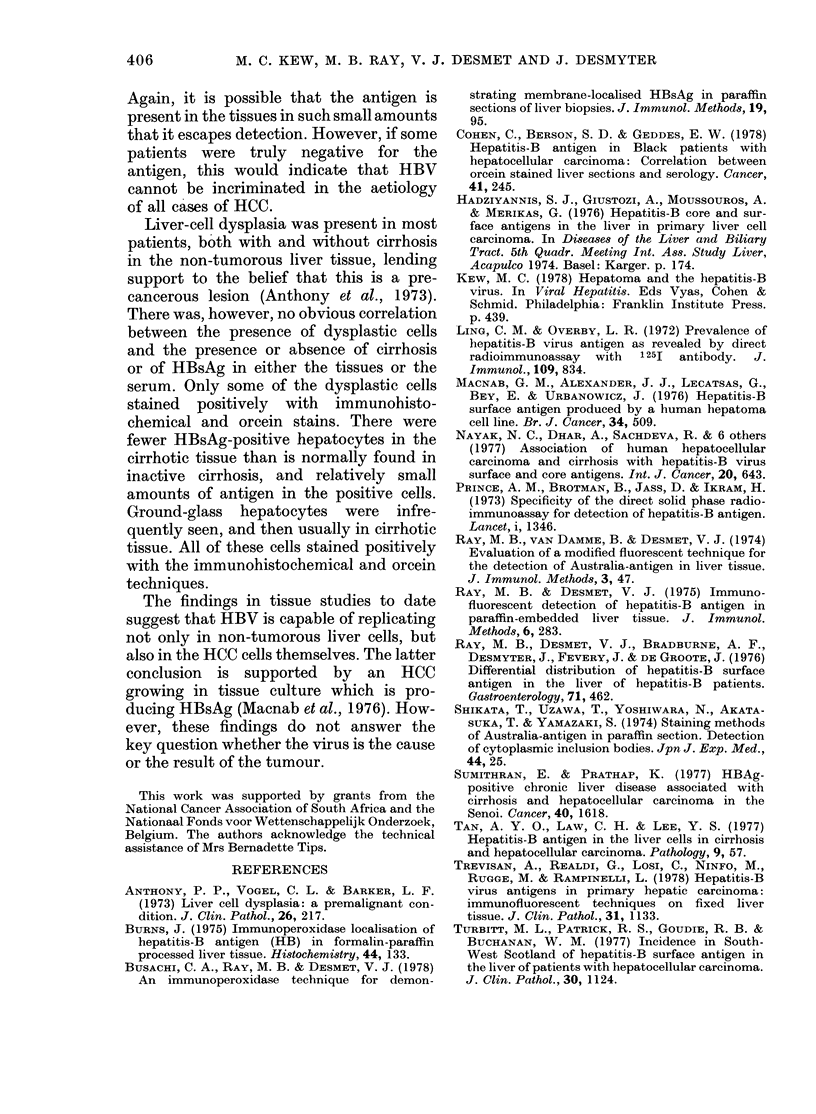# Hepatitis-B surface antigen in tumour tissue and non-tumorous liver in black patients with hepatocellular carcinoma.

**DOI:** 10.1038/bjc.1980.63

**Published:** 1980-03

**Authors:** M. C. Kew, M. B. Ray, V. J. Desmet, J. Desmyter

## Abstract

**Images:**


Br. J. Cancer (1980) 41, 399

### HEPATITIS-B SURFACE ANTIGEN IN TUMOUR TISSUE AND NON-TUMOROUS LIVER IN BLACK PATIENTS WITH HEPATOCELLULAR CARCINOMA

M. C. KEW, M. B. RAY, V. J. DESMET AND J. DESMYTER

*From the Department of Medicine, University of Witwatersrand and Johannesburg Hospital, Johannesburg, South Africa, and the Laboratory of Histochemistry and Cytochemistry and Department of Virology, Akademische Ziekenhuis Sint Rafael, University of Leuven, Belgium*

Received 6 September 1979 Accepted 29 October 1979

**Summary.—Formalin-fixed, paraffin-embedded sections of liver and tumour tissue obtained at necropsy from 44 southern African Blacks with hepatocellular carcinoma were stained for hepatitis-B virus surface antigen by immunofluorescence, immunoperoxidase and orcein techniques. The antigen was present in the serum of 68% of the patients. Staining for tissue antigen was positive in 45% of the patients. Non-tumorous hepatocytes alone stained positively in 22·5% of patients, tumour cells alone in 12·5% and both in 10%. Antigen was present in relatively few tumour cells and the amounts detected were small; it was more readily detectable in moderately differentiated than in poorly differentiated malignant cells. Identical results were obtained with immunofluorescence and immunoperoxidase staining, but the orcein stain failed to demonstrate the antigen in tumour cells. Cirrhosis was present in the non-tumorous liver in 70% of the patients. Antigen was detected in cirrhotic tissue in 43% of the patients with cirrhosis, and in non-tumorous liver tissue in 8% of those without cirrhosis, but this difference was not significant. The antigen frequency in tumour tissue was the same in patients with and without cirrhosis. No correlation was found between the presence of liver-cell dysplasia and the presence or absence of either the antigen or cirrhosis in the non-tumorous liver tissue. Ground-glass hepatocytes were seen in non-tumorous liver tissue of 5 patients, but not in tumour tissue. While 54% of the patients with antigenaemia had demonstrable tissue antigen, 10% of patients with tissue antigen had no detectable antigenaemia.**

WHILE THERE IS undoubtedly a close association between chronic hepatitis-B virus (HBV) infection and hepatocellular carcinoma (HCC), the nature of the association has not been elucidated, and there is as yet no proof that the virus causes the tumour. Antigenic markers of HBV are frequently found in the serum of patients with HCC, particularly in those parts of the world where this tumour is common (Kew, 1978). Detection of these markers in the non-tumorous liver tissue, whether this is normal or cirrhotic, and especially in the malignant cells themselves, might provide more direct evidence for an oncogenic role of the virus. Some information in this regard is already available (Tan *et al.*, 1977; Nayak *et al.*, 1977; Turbitt *et al.*, 1977; Trevisan *et al.*, 1978). Cohen *et al.* (1978) reported positive orcein staining of paraffin sections of non-tumorous liver tissue in 36% and of tumour cells in 6% of southern African Blacks with hepatocellular carcinoma. The purpose of the present study was to confirm and extend the latter findings,

Correspondence and reprint requests to: Professor M. C. Kew, Department of Medicine, University of the Witwatersrand Medical School, Hospital Hill, Johannesburg 2001, South Africa.

using specific immunofluorescence and immunoperoxidase techniques in addition to orcein staining.

### MATERIALS AND METHODS

*Patients studied.*—The study was based on 44 southern African Blacks with HCC. These patients were chosen because their serum HBsAg status was known, and a necropsy was performed. They were all males, their ages at death ranging from 19 to 60 years, with a mean of 36 years. Sixty per cent of the patients were Shangaans from Mozambique, while the remainder came from Malawi, Transkei, Botswana, Zululand, Lesotho and rural South Africa. Most of the patients had received some form of cancer chemotherapy at some time.

*Tissue studies.*—Blocks of HCC tissue and non-tumorous liver tissue, each measuring at least 1 $cm^2$, were obtained at necropsy, fixed in formalin and embedded in paraffin in the usual way. Unstained sections mounted on glass slides were coded and sent to Leuven for examination after special staining. In almost all cases two slides per patient were studied. The patients' serum HBsAg status was not made known to the histopathologists responsible for examining the slides until after completion of the study. In addition to being prepared for routine histological examination, all sections were stained with the immunofluorescence, immunoperoxidase and orcein techniques. The methods used have previously been described in detail: immunofluorescence (Ray & Desmet, 1975); peroxidase and anti-peroxidase (Burns, 1975; Busachi *et al.*, 1978); orcein staining (Shikata *et al.*, 1974). For control purposes, a known positive and a known negative slide were included in each batch of slides stained. The specificity of all positive staining reactions was checked, as described in previous publications (Ray *et al.*, 1974; Busachi *et al.*, 1978). In each case the presence or absence of cirrhosis, liver cell dysplasia and ground-glass hepatocytes was determined on sections stained with haematoxylin and eosin. Dysplastic cells were identified according to the criteria of Anthony *et al.* (1973). The grading system (0–4+) was based on the estimated overall percentage of dysplastic cells in the non-tumorous liver tissue: 0: absent; +: 25%; 2+: 25–50%; 3+: 50–75%; 4+: 75–100%.

*Serum studies.*—HBsAg was detected in the patients' sera by solid-phase radioimmunoassay, using Ausria II–$^{125}$I (Abbot Laboratories) (Ling & Overby, 1972). All positive results were confirmed with the neutralizing-antibody technique described by Prince *et al.* (1973).

### RESULTS

### *HBsAg in serum*

HBsAg was detected in the sera of 30 of the 44 patients (68·2%; Table I). Of the 26 patients with HBs antigenaemia, and in whom both tumour and liver tissue were present on the slides examined, 14 (54%) had evidence of HBsAg in the non-tumorous liver and/or tumour tissue. Fourteen of the 18 patients with positive tissue staining had HBs antigenaemia. Thus, in 4 patients (10%) HBsAg was demonstrable in tissue but not in serum.

### *HBsAg in tissue*

In 4 of the patients only tumour tissue was present on the slides examined. Evidence of tissue HBsAg was consistently found in 18 patients (45%; Table I). The tissue findings with the immunofluorescence and immunoperoxidase techniques were identical, but overall fewer positive results (13/40; 32·5%) were obtained with the orcein stain (Table II). This was entirely due to the fact that in none of the slides did the tumour cells stain positively with orcein. Of the 18 patients with tissue HBsAg, the non-tumorous hepatocytes alone were positive in 9 (22·5% of whole group), the tumour cells alone were positive in 5 (12·5%), and both were positive in 4 (10%; Tables I and II). Twenty-eight of the 40 patients (70%) had cirrhosis in the non-tumorous liver tissue. This was usually of the macronodular type, though in some cases it was a mixed macronodular-micronodular cirrhosis. The distribution of HBsAg in the tissues of the patients with and without cirrhosis is shown in Table II. Twelve of those with cirrhosis (43%) had HBsAg in the cirrhotic tissue (Fig. 1), while one of the 12 patients with-

TABLE I.—*Detailed results in individual patients of HBsAg in tissues and serum and of liver-cell dysplasia and ground-glass cells*

| Patient | Tissue HBsAg: Non-tumorous liver | Tissue HBsAg: HCC tissue | Ground-glass hepatocytes | Dysplastic cells | Serum HBsAg |
|---|---|---|---|---|---|
| HCC + cirrhosis | | | | | |
| 1 | − | − | − | + | + |
| 2 | − | − | − | + | + |
| 3 | − | + | − | + | + |
| 4 | + | − | − | 0 | + |
| 5 | + | − | + | + | + |
| 6 | + | − | − | + | + |
| 7 | + | + | − | + | + |
| 8 | − | − | − | 0 | + |
| 9 | − | + | − | + − + + | + |
| 10 | − | − | − | + + | − |
| 11 | + | − | − | 0 | − |
| 12 | + | − | − | 0 | + |
| 13 | − | − | − | + | − |
| 14 | − | − | − | + − + + | + |
| 15 | − | − | − | 0 | + |
| 16 | − | + | − | + | − |
| 17 | − | − | − | + | + |
| 18 | + | − | − | + | + |
| 19 | + | − | + | + + | + |
| 20 | − | − | − | + + | − |
| 21 | − | − | − | + | + |
| 22 | + | − | − | + | − |
| 23 | − | − | − | + | + |
| 24 | − | − | − | + | + |
| 25 | + | + | + | + | + |
| 26 | − | − | − | + | − |
| 27 | + | + | − | + | + |
| 28 | + | − | + | + + | + |
| HCC No cirrhosis | | | | | |
| 29 | − | + | − | + + | − |
| 30 | − | − | − | + | − |
| 31 | − | − | − | 0 | + |
| 32 | − | − | − | + | − |
| 33 | − | + | − | + | + |
| 34 | − | − | − | + + | + |
| 35 | − | − | − | 0 | + |
| 36 | − | − | − | 0 | − |
| 37 | − | − | − | + | − |
| 38 | − | − | − | + + | − |
| 39 | + | + | + | + | + |
| 40 | − | − | − | + | − |
| HCC tissue only | | | | | |
| 41 | | − | | | + |
| 42 | | − | | | + |
| 43 | | − | | | + |
| 44 | | − | | | + |

out cirrhosis (8·3%) had positive non-tumorous hepatocytes (Table II). This difference is not significant using the Yates modification of the $\chi^2$ test. Six of the patients with cirrhosis (21·4%) had HBsAg in tumour tissue (Fig. 2), while 3 of those without cirrhosis (25%) had positive tumour tissue (Table II).

*Cellular distribution*

*Non-tumorous tissue.*—In cirrhotic nodules, positively staining cells were frequently found in groups along the fibrous septa. The remaining areas were either entirely negative or showed a few scattered positive cells. The number of HBsAg-containing hepatocytes was much lower than that usually found in liver tissue from patients with relatively inactive cirrhosis (Ray *et al.*, 1976). At an intracellular level HBsAg showed mostly a focal cytoplasmic distribution.

*HCC tissue.*—Only a small number of tumour cells were positive for HBsAg. Furthermore, the amount of antigen in the cells was small. HBsAg was found only in the perinuclear area. The antigen was more readily detectable in moderately differentiated than in poorly differentiated malignant cells.

*Liver-cell dysplasia*

This was looked for in the 40 patients in whom non-tumorous liver tissue was present on the slides. Of these, 28 had cirrhosis in the non-tumorous liver tissue. HBsAg was demonstrable in the cirrhotic tissue in 15 of the 28 patients, and 12 of these had dysplastic cells (Fig. 3). These cells were also present in 11 of the 13 patients in whom HBsAg was not seen in the cirrhotic tissue. Of the 12 patients without cirrhosis, dysplastic cells were seen in all 3 in whom the tissue was positive for HBsAg and in 6/9 without positive staining. Thus, no correlation was observed between the presence of dysplastic cells and the presence or absence of either HBsAg or cirrhosis in the non-tumorous liver tissue. There was no definite pattern with regard to staining of individual dysplastic cells with immunofluorescence, immunoperoxidase or orcein stains; some of the cells stained positively while others did not. No correlation was

TABLE II.—*Comparison of the results obtained by immunohistochemistry and orcein staining in HCC patients with and without cirrhosis in the non-tumorous liver tissue*

| Histological diagnosis | | Total positivity | IF Non-tumour tissue | IF Tumour tissue | IF Both | PAP Non-tumour tissue | PAP Tumour tissue | PAP Both | Orcein Non-tumour tissue | Orcein Tumour tissue | Orcein Both | Serum HBsAg Pos. | Serum HBsAg Neg. |
|---|---|---|---|---|---|---|---|---|---|---|---|---|---|
| HCC | | | | | | | | | | | | | |
| (a) cirrhosis | 28 | 15 | 9 | 3 | 3 | 9 | 3 | 3 | 12 | — | — | 21 | 7 |
| (b) without cirrhosis | 12 | 3 | — | 2 | 1 | — | 2 | 1 | 1 | — | — | 5 | 7 |
| Total | 40 | 18 | 9 | 5 | 4 | 9 | 5 | 4 | 13 | — | — | 26 | 14 |

FIG. 1.—Hepatocellular carcinoma with cirrhosis. HBsAg is demonstrated as dark brown deposits in the cytoplasm of a group of non-tumorous hepatocytes. Orcein stain ( × 320).

FIG. 2.—Hepatocellular carcinoma with cirrhosis. Indirect immunofluorescence staining. HBsAg is demonstrated in small amounts in different cytoplasmic locations in cancerous hepatocytes. In the same area, PAP and orcein stainings gave strong non-specific background staining ( × 320).

FIG. 3.—Demonstrates an area with many dysplastic hepatocytes. Some of them show HBsAg positivity. Orcein stain ( × 512).

found between the distribution of dysplastic cells and lobular topography in non-cirrhotic liver tissue. However, in the cirrhotic nodules dysplastic cells were mostly found at the periphery of the nodules. Liver cell dysplasia was present in 19/24 patients (79%) with and 12/14 (96%) without HBs antigenaemia.

*Ground-glass hepatocytes*

Ground-glass cells were visualized in non-cirrhotic liver tissue in 1 patient and in cirrhotic tissue of 4 patients. All of these cells stained positively for HBsAg with both immunohistochemical and orcein stains. No ground-glass cells were observed in tumour tissue.

### DISCUSSION

Our findings agree, in the main, with those of Cohen *et al.* (1978). The studies were performed on similar patient populations, having nearly the same prevalence of HBs antigenaemia, and differed only in so far that we used immunospecific techniques in addition to the orcein stain for detecting HBsAg, whereas the earlier investigation was confined to orcein staining. HBsAg was detected in the cytoplasm of non-tumorous hepatocytes with equal frequency (32·5 and 36%) in the two studies. However, we found the cytoplasm of the tumour cells to stain positively for the antigen significantly more often than Cohen *et al.* (1978) (22·5 as against 6%). A possible explanation for this difference is the relative insensitivity of the orcein stain. This is suggested by our finding that the 9 patients in whom tumour cells stained positively for HBsAg with both immunofluorescence and immunoperoxidase failed to do so with orcein, and that when the antigen was detected in these cells only small amounts were present. With the greater quantities of HBsAg in non-malignant hepatocytes, we found an excellent correlation between the staining techniques. In a previous study from this laboratory of patients with HBsAg-related liver disease other than HCC, immunofluorescence was found to be more sensitive than orcein staining in detecting the antigen (Ray *et al.*, 1974). This observation was confirmed recently when Turbitt *et al.* (1977) found orcein staining less sensitive than the immunoperoxidase technique in detecting HBsAg in hepatocellular carcinoma and cirrhosis. However, in the study of Nayak *et al.* (1977) none of the specimens which failed to stain with orcein gave positive reactions with immunoperoxidase.

When patients from those parts of the world where HCC and the HBV carrier state occur commonly have been studied, high positivity rates of HBsAg in tissue have been recorded (Hadziyannis *et al.*, 1976; Nayak *et al.*, 1977; Tan *et al.*, 1977; Sumithran & Prathap, 1977). By contrast, in Scotland, where both HCC and chronic HBs antigenaemia are infrequent, only 10% of tissues stained positively for HBsAg (Turbitt *et al.*, 1977). The prevalence of tissue HBsAg in southern African Blacks with this tumour lies between the two extremes, which is intriguing because the prevalence of HBs antigenaemia is higher in the African patients than in most or all of the other HCC populations studied (Kew, 1978). The frequency with which HBsAg is detected in tissue increases with the adequacy of the sample (Nayak *et al.*, 1977). Both of the studies in southern African Blacks were conducted on adequate or reasonably adequate necropsy specimens. Even with a single block of tissue, Nayak *et al.* (1977) found a positivity rate of 75%, which is appreciably greater than the figure of ~40% in southern African Blacks. One possible explanation for this discrepancy might be inter-study differences in the prevalence of cirrhosis in the non-tumorous liver. Indeed, cirrhosis was present in 92% of the patients in whom HBsAg was found most often (94%) in the non-tumorous liver (Nayak *et al.*, 1977). However, the prevalence of cirrhosis in the other studies was not dissimilar. Furthermore, HBsAg has also been consistently detected in non-cirrhotic liver tissue of HCC patients.

In previous studies, HBsAg has rarely been detected in tumour cells, the highest incidence reported being the 8% found by Nayak *et al.* (1977) in Indian patients. It seems that the southern African Black with HCC may be more likely than other patients with this tumour to have the antigen demonstrable in malignant hepatocytes (22·5%). As was the case in other studies (Hadziyannis *et al.*, 1976; Nayak *et al.*, 1977; Turbitt *et al.*, 1977) when HBsAg was demonstrable in malignant hepatocytes, the quantities were small. The antigen had a different distribution in the tumour cells, occupying a perinuclear position. It was more easily detectable in moderately differentiated than in poorly differentiated tumours, confirming previous observations (Nayak *et al.*, 1977; Trevisan *et al.*, 1978). This observation, together with the finding of HBsAg predominantly in the periphery of tumour nodules, suggest that virus infection of neoplastic cells may be an expression of variable metabolic environments within uniform cell clones. An alternative explanation for the peripheral distribution of the antigen is that normal hepatocytes may become enmeshed in the growing edge of the tumour, and it is these cells which take up the stain.

As in previous studies, a few patients without apparent HBs antigenaemia had the antigen demonstrable in liver or tumour tissue. This finding might be explained by the antigen being present in the patient's serum in too small an amount to be detectable with present-day laboratory techniques. Alternatively, it is possible that, for some reason, HBsAg in tissue is not released into the blood. The opposite picture (*i.e.* HBs antigenaemia without detectable antigen in the tissues) was more frequently seen. Examining further blocks of tissue would increase the tissue positivity rate somewhat, but negative cases would undoubtedly remain.

Again, it is possible that the antigen is present in the tissues in such small amounts that it escapes detection. However, if some patients were truly negative for the antigen, this would indicate that HBV cannot be incriminated in the aetiology of all cases of HCC.

Liver-cell dysplasia was present in most patients, both with and without cirrhosis in the non-tumorous liver tissue, lending support to the belief that this is a pre-cancerous lesion (Anthony *et al.*, 1973). There was, however, no obvious correlation between the presence of dysplastic cells and the presence or absence of cirrhosis or of HBsAg in either the tissues or the serum. Only some of the dysplastic cells stained positively with immunohisto-chemical and orcein stains. There were fewer HBsAg-positive hepatocytes in the cirrhotic tissue than is normally found in inactive cirrhosis, and relatively small amounts of antigen in the positive cells. Ground-glass hepatocytes were infre-quently seen, and then usually in cirrhotic tissue. All of these cells stained positively with the immunohistochemical and orcein techniques.

The findings in tissue studies to date suggest that HBV is capable of replicating not only in non-tumorous liver cells, but also in the HCC cells themselves. The latter conclusion is supported by an HCC growing in tissue culture which is pro-ducing HBsAg (Macnab *et al.*, 1976). However, these findings do not answer the key question whether the virus is the cause or the result of the tumour.

This work was supported by grants from the National Cancer Association of South Africa and the Nationaal Fonds voor Wettenschappelijk Onderzoek, Belgium. The authors acknowledge the technical assistance of Mrs Bernadette Tips.

### REFERENCES

Anthony, P. P., Vogel, C. L. & Barker, L. F. (1973) Liver cell dysplasia: a premalignant condition. *J. Clin. Pathol.*, **26**, 217.

Burns, J. (1975) Immunoperoxidase localisation of hepatitis-B antigen (HB) in formalin-paraffin processed liver tissue. *Histochemistry*, **44**, 133.

Busachi, C. A., Ray, M. B. & Desmet, V. J. (1978) An immunoperoxidase technique for demonstrating membrane-localised HBsAg in paraffin sections of liver biopsies. *J. Immunol. Methods*, **19**, 95.

Cohen, C., Berson, S. D. & Geddes, E. W. (1978) Hepatitis-B antigen in Black patients with hepatocellular carcinoma: Correlation between orcein stained liver sections and serology. *Cancer*, **41**, 245.

Hadziyannis, S. J., Giustozi, A., Moussouros, A. & Merikas, G. (1976) Hepatitis-B core and surface antigens in the liver in primary liver cell carcinoma. In *Diseases of the Liver and Biliary Tract. 5th Quadr. Meeting Int. Ass. Study Liver, Acapulco* 1974. Basel: Karger. p. 174.

Kew, M. C. (1978) Hepatoma and the hepatitis-B virus. In *Viral Hepatitis*. Eds Vyas, Cohen & Schmid. Philadelphia: Franklin Institute Press. p. 439.

Ling, C. M. & Overby, L. R. (1972) Prevalence of hepatitis-B virus antigen as revealed by direct radioimmunoassay with $^{125}$I antibody. *J. Immunol.*, **109**, 834.

Macnab, G. M., Alexander, J. J., Lecatsas, G., Bey, E. & Urbanowicz, J. (1976) Hepatitis-B surface antigen produced by a human hepatoma cell line. *Br. J. Cancer*, **34**, 509.

Nayak, N. C., Dhar, A., Sachdeva, R. & 6 others (1977) Association of human hepatocellular carcinoma and cirrhosis with hepatitis-B virus surface and core antigens. *Int. J. Cancer*, **20**, 643.

Prince, A. M., Brotman, B., Jass, D. & Ikram, H. (1973) Specificity of the direct solid phase radio-immunoassay for detection of hepatitis-B antigen. *Lancet*, i, 1346.

Ray, M. B., van Damme, B. & Desmet, V. J. (1974) Evaluation of a modified fluorescent technique for the detection of Australia-antigen in liver tissue. *J. Immunol. Methods*, **3**, 47.

Ray, M. B. & Desmet, V. J. (1975) Immuno-fluorescent detection of hepatitis-B antigen in paraffin-embedded liver tissue. *J. Immunol. Methods*, **6**, 283.

Ray, M. B., Desmet, V. J., Bradburne, A. F., Desmyter, J., Fevery, J. & de Groote, J. (1976) Differential distribution of hepatitis-B surface antigen in the liver of hepatitis-B patients. *Gastroenterology*, **71**, 462.

Shikata, T., Uzawa, T., Yoshiwara, N., Akatasuka, T. & Yamazaki, S. (1974) Staining methods of Australia-antigen in paraffin section. Detection of cytoplasmic inclusion bodies. *Jpn J. Exp. Med.*, **44**, 25.

Sumithran, E. & Prathap, K. (1977) HBAg-positive chronic liver disease associated with cirrhosis and hepatocellular carcinoma in the Senoi. *Cancer*, **40**, 1618.

Tan, A. Y. O., Law, C. H. & Lee, Y. S. (1977) Hepatitis-B antigen in the liver cells in cirrhosis and hepatocellular carcinoma. *Pathology*, **9**, 57.

Trevisan, A., Realdi, G., Losi, C., Ninfo, M., Rugge, M. & Rampinelli, L. (1978) Hepatitis-B virus antigens in primary hepatic carcinoma: immunofluorescent techniques on fixed liver tissue. *J. Clin. Pathol.*, **31**, 1133.

Turbitt, M. L., Patrick, R. S., Goudie, R. B. & Buchanan, W. M. (1977) Incidence in South-West Scotland of hepatitis-B surface antigen in the liver of patients with hepatocellular carcinoma. *J. Clin. Pathol.*, **30**, 1124.